# Epigenetic Therapies in Endocrine-Related Cancers: Past Insights and Clinical Progress

**DOI:** 10.3390/cancers17152418

**Published:** 2025-07-22

**Authors:** Dhruvika Varun, Maria Haque, Jorja Jackson-Oxley, Rachel Thompson, Amber A. Kumari, Corinne L. Woodcock, Anna E. Harris, Srinivasan Madhusudan, Emad Rakha, Catrin S. Rutland, Nigel P. Mongan, Jennie N. Jeyapalan

**Affiliations:** 1 School of Veterinary Medicine and Sciences, Faculty of Medicine and Health Sciences, University of Nottingham, Nottingham LE12 5RD, UK; 2 Biodiscovery Institute, Faculty of Medicine and Health Sciences, University of Nottingham, Nottingham NG7 2RD, UK; 3 Department of Pharmacology, Weill Cornell Medicine, New York, NY 10021, USA

**Keywords:** breast cancer, ovarian cancer, thyroid cancer, prostate cancer, endometrial cancer, clinical trials, pharmacological inhibitors

## Abstract

In hormone-dependent cancers, front-line treatment options include surgery and therapies that target hormone dependance. These therapies are effective initially but fail in tumors that recur, develop resistance or present at an advanced stage. Consequently, new therapeutic avenues are urgently needed. Increasing evidence implicates epigenetic modulators in tumor initiation, progression and therapeutic response, making them attractive biomarkers for patient stratification and targets for intervention. Over the past two decades, the discovery and development of small-molecule inhibitors directed against key epigenetic regulators have accelerated. This review provides a comprehensive overview of the major epigenetic targets, the inhibitors developed against them and the clinical trials currently underway in endocrine-related cancers. While epigenetic agents have shown limited benefits as monotherapies, their use in combination regimens is emerging as a strategy to overcome resistance and enhance the efficacy of existing treatments. We summarize the current landscape of combination trials, highlight early signs of clinical activity and discuss the opportunities and challenges inherent in integrating epigenetic drugs into the management of advanced endocrine-related cancers.

## Simple Summary

Current treatment options in endocrine-related cancers target vulnerabilities within these cancers such as hormone dependency, which completely cures the disease or prevents advancement. Over time, cancers recur or become resistant to these front-line treatment options. A biological mechanism that does not alter the DNA sequence but can influence which genes are on/off within the cell is known as epigenetics. A single factor in the epigenetic machinery can alter how cancers react to treatment by influencing regulatory systems within the cancer cells, allowing them to adapt. This review article explores current clinical trials, detailing the successes and challenges of utilizing epigenetic drugs in combination treatments. Epigenetic drugs show promise as a future therapeutic avenue, but obstacles such as adverse effects must be tackled to make these drugs a new tool in the fight against cancer.

## 1. Introduction

Non-mutational epigenetic reprogramming has now emerged as a hallmark of cancer [1]. There is evidence of aberrant DNA methylation, histone modifications and RNA modifications, all playing a role in cancer development and progression (reviewed in [2]).

Endocrine-related cancers including breast, prostate, ovarian, endometrial and thyroid cancers rely on steroid or peptide hormones for their growth and progression. Thus, these are often called hormone-dependent cancers [3]. About 75% of breast cancers (BCa) are estrogen receptor alpha (ER)-positive and driven by estrogen signaling [4]. Thus, endocrine therapy is the preferred treatment for these cancers, which commonly includes estrogen biosynthesis blockers like aromatase inhibitor agonists, selective ER modulators like tamoxifen and ER antagonists like fulvestrant [5,6]. However, resistance to endocrine therapy can occur through several mechanisms, one of which is a loss of ER/progesterone receptor (PR) expression, promoting the hormone-independent survival and growth of tumor cells [7,8]. Epigenetic mechanisms play a role in silencing *ESR1* and *PGR* genes in BCa, which encode ER and PR, respectively. The *ESR1* promoter is hypermethylated in BCa by the DNMT3B/ZEB1/HDAC1 complex, resulting in transcriptional silencing [9]. Additionally, increased activity of the HDAC1-containing NuRD complex is associated with transcriptional repression of *ESR1* in BCa [10]. Polycomb repressors and HDAC enzymes bind the *PGR* promoter to initiate transcriptional repression in the absence of ER signaling, followed by the recruitment of DNA methyltransferases (DNMTs) to methylate adjacent CpG sites, resulting in long-term suppression of PR expression [11].

The balance between estrogen and progesterone is a critical factor in the development of ovarian cancer (OvCa) and endometrial cancer (ECa). While estrogen is associated with an increased proliferation and metastatic potential of cells, progesterone has antiproliferative and anti-inflammatory effects on ovarian and endometrial cells [12,13,14,15]. Increased estrogen biosynthesis and reduced progesterone levels in women are strongly associated with OvCa and ECa incidence and can even predict outcomes [14,16]. Paclitaxel- and platinum-based chemotherapy is the first-line treatment for OvCa, but immunotherapy and poly (ADP-ribose) polymerase (PARP) inhibitors have also emerged as effective treatment options [17]. Drug resistance unfortunately occurs in approximately 75% of patients with advanced OvCa within three years of treatment initiation [18]. Epigenetic modifications can regulate drug resistance in OvCa by upregulating drug efflux- and metabolism-related proteins, upregulating bypass signaling pathways like Notch signaling, increasing anti-apoptotic protein expression and pro-survival signaling, and maintaining cancer stem cell (CSC) survival [19]. One of the most effective treatment options for advanced or recurrent ECa is progestin (synthetic progesterone) therapy to activate PR signaling and counteract the elevated estrogen signaling [20,21]. However, resistance to progestin therapy often occurs, primarily due to the decline or loss of PR expression in endometrial tumors, leading to poor prognosis [22]. DNA hypermethylation is observed in promoters of tumor suppressor genes (TSGs) like *MLH1*, *PTEN*, *MGMT*, *RASSF1A* and *CDH1* in early-stage ECa, promoting carcinogenesis [23]. Additionally, the *PGR* promoter region is hypermethylated in advanced ECa, which contributes to PR loss and treatment resistance [24]. HDAC1, 2 and 3 are overexpressed in ECa tissue compared to non-malignant tissue and can regulate various cellular functions including proliferation, apoptosis and differentiation [25,26]. Histone methylation marks H3K4me2/3 are globally higher in ECa tissue than the normal endometrium, along with the overexpression of lysine methyltransferases (KMTs) like EZH2, EHMT2/G9a, NSD2 and ASH2L and the lysine demethylase (KDM) KDM1A/LSD1 [27,28]. Aberrant histone methylation by these epigenetic proteins is linked with poor ECa prognosis, the clinicopathological disease grade and the regulation of cell proliferation and invasion [28].

Prostate cancer (PCa) is driven by androgen receptor (AR) signaling [29]. The main androgen in people is testosterone, which is converted to 5α-dihydrotestosterone and binds to AR to initiate the AR signaling cascade [30]. Thus, androgen deprivation therapies (ADTs) like gonadotropin-releasing hormone (GnRH) analogs or AR-targeting antiandrogens like bicalutamide and enzalutamide are primary treatment options for PCa [31]. However, PCa tumors often become resistant to ADT within approximately 24 months of treatment, leading to the emergence of castration-resistant prostate cancer (CRPC), which is more challenging to treat [29]. Epigenetic and epitranscriptomic mechanisms play a role in both PCa tumorigenesis and ADT resistance [32,33]. Increased promoter methylation is observed on PCa-associated TSGs like *GSTP1*, *RARβ*, *RUNX3*, *HOXD3* and *BMP7* in PCa tissue as compared to non-malignant tissue, suggesting a potential role in tumorigenesis [34,35,36]. Histone acetyltransferases (HATs), such as CBP/p300, are involved in transcriptional activation of the AR target gene *KLK3*, and are overexpressed in CRPC as compared to primary adenocarcinomas, suggesting a potential role in ADT resistance [36,37]. KDMs like KDM3A, KDM4B/C and KDM1A act as AR coactivators, which function by demethylating H3K9/K4, leading to transcriptional activation of AR target genes [38,39,40]. KDM1A and KDM5B have also been shown to regulate AR expression in CRPC cell lines [32]. Enhancer of zeste homolog 2 (EZH2), a member of the polycomb repressive complex 2 (PRC2), is an important H3K27 KMT in PCa. However, in CRPC, it works independently of PRC2, as an AR coactivator [41]. EZH2 inhibition has been shown to resensitize CRPC cells and xenografts to enzalutamide [42].

Thyroid cancer (TCa) is the most common endocrine cancer, with a 3–5-fold higher prevalence in females than in males [43]. Thyroid hormones (THs) and thyroid-stimulating hormone (TSH) both regulate the development of thyroid follicular cells and are strongly associated with TCa recurrence and invasive clinicopathological features [44]. Additionally, estrogen and androgen can also regulate TCa development through complex genomic and non-genomic pathways [43]. In well-differentiated TCa cells, sodium iodide symporter (NIS), thyroglobulin (Tg) and thyroperoxidase (TPO) mediate the uptake and accumulation of iodide [45,46]. Radioactive-iodine (RAI) therapy is a common treatment option for TCa which delivers targeted radiation to TCa cells, which ultimately causes cell death [47]. The expression of thyroid markers is downregulated in poorly differentiated TCa cells, resulting in reduced uptake of RAI therapy, eventually rendering these cancers unresponsive to RAI [48]. Genes involved in thyroid differentiation and iodine uptake such as *NIS*, *TSHR* (TSH receptor), *pendrin* (sodium-independent chloride/iodide transporter), *SL5A8* (solute carrier family 5 member 8) and *TTF-1* (thyroid transcription factor-1) are often silenced due to DNA hypermethylation in TCa [49]. There is also a global reduction in histone acetylation during the transition of TCa from differentiated to undifferentiated tumors [50]. Histone acetylation in TCa cells was also regulated by TSH stimulation, thus potentially playing a role in TCa development, progression and RAI resistance [50].

**Figure 1:**
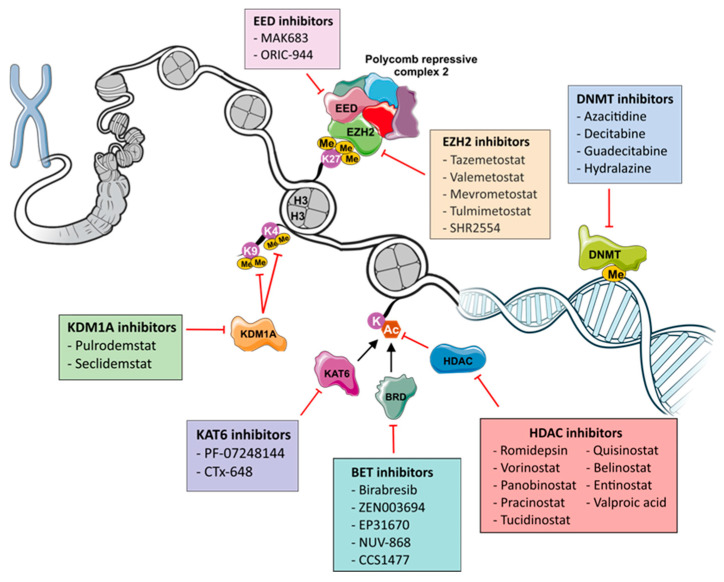
Schematic overview of epigenetic drugs and their targets that have been trialed in endocrine-related cancers. Created using NIAD NIH BIOART and Bioicons.

The evidence to date shows that epigenetic modifications play a crucial role in the development, progression and treatment resistance of endocrine-related cancers. Since epigenetic modifications are reversible, there is a growing interest in exploring the efficacy of epigenetic drugs in reversing the oncogenic epigenetic changes in cancer cells, therefore forming an attractive treatment strategy [51]. In this review, we discuss past and ongoing research on the safety and efficacy of a range of epigenetic drugs (Figure 1) in treating endocrine-related cancers as monotherapies or in combination with existing treatments.

## 2. Epigenetic Drugs Being Trialed in Endocrine-Related Cancers

### 2.1. DNA Methyltransferase Inhibitors

DNA methyltransferase inhibitors (DNMTi prevent the methyltransferase activity of DNMTs, predominantly DNMT1, thus reducing hypermethylation on target gene promoters (Table 1) [52]. Azacitidine and decitabine are cytidine analogs and were the first DNMT inhibitors to be Food and Drug Administration (FDA)-approved in 2004 and 2006, respectively, for treating hematological malignancies [52,53]. They are currently being investigated for the treatment of solid tumors [52]. Azacitidine, within the cell, is phosphorylated by uridine cytidine kinase and converted into its active form 5′-azacytidine-5′-triphosphate which, as a ribonucleoside, is integrated into the RNA and to some extent in the DNA backbone of proliferating cells [54,55]. The conversion of ribonucleotides to deoxyribonucleotides is facilitated by the ribonucleotide reductase enzyme, which is often a rate-limiting step with azacitidine treatment [54]. In the DNA, the C-5 of the cytosine ring is replaced with an N-5 of azacytosine, which blocks DNA methylation due to the formation of azacytosine–guanine dinucleotides that disrupt the interaction between the DNA and DNMTs, trap DNMT1 and subsequently lead to its depletion [55,56]. Decitabine has a similar mechanism of action to azacitidine. However, it is converted to 5-aza-2′-deoxycytidine-5′-triphosphate by deoxycytidine kinase which, as a deoxyribose analog, is incorporated exclusively in the DNA [54,55]. Thus, decitabine is 30 times more potent than azacitidine [55]. At high decitabine concentrations, DNMT trapping triggers a DNA damage response which can induce cell death; however, at lower concentrations the drug can covalently bind and inactivate DNMTs without inducing cell death [57]. A limitation of decitabine is that it can undergo rapid deamination by cytidine deaminase in the liver, which can cause granulocytopenia, thus necessitating the optimization of its clinical dosage and pharmacodynamic/pharmacokinetic evaluation to avoid side effects [57].

Guadecitabine is a next-generation DNMTi which is a dinucleotide containing decitabine linked to deoxyguanosine by a phosphodiester bond [58]. Unlike decitabine, guadecitabine is not prone to deamination by cytidine deaminase [58]. The cleavage of the phosphodiester bond by phosphorylase leads to the gradual release of decitabine, thus prolonging the half-life and exposure time of the drug and enhancing its activity [58,59]. Additionally, the reduced maximum plasma concentration may help avoid dose-related toxicities [59]. Guadecitabine has shown good safety and efficacy in treating leukemia patients and is currently being investigated in solid tumors [60,61].

Hydralazine, although initially identified as a smooth-muscle relaxant, was subsequently shown to reduce DNA methylation in systemic lupus erythematosus, thus acting as a DNMTi [62]. It binds the active site of DNMT1 and DNMT3A, thereby inactivating their methyltransferase activity [62]. Hydralazine in combination with magnesium valproate has shown promising clinical results in treating chemotherapy-resistant solid tumors [63].

More recently, the development of DNMT1 degraders/inhibitors GSK3685032 [64] and GSK-3484862 [65] has shown the reversible depletion of DNMT1 activity and hypomethylation, with greater tolerability in vivo observed for GSK3685032 compared to decitabine, identifying new compounds for clinical use [64].

### 2.2. Histone Acetyltransferase (HAT) and Bromodomain and Extraterminal (BET) Inhibitors

HATs acetylate lysines on histones H3 and H4, which opens chromatin, allowing for DNA replication, transcriptional activation and DNA repair [66]. The development of inhibitors to HATs has faced challenges, but several inhibitors are currently being trialed (Table 1) [67]. One HAT that has shown promise as a drug target is the lysine acetyltransferase 6 (KAT6), which acetylates histone 3 lysine 23 (H3K23ac) [68]. The selective KAT6A/B inhibitor (KAT6i), PF-07248144, is currently undergoing a phase I study to evaluate its safety and efficacy, following PF-07248144 monotherapy and use in combination with fulvestrant in solid tumors including PCa and BCa (NCT04606446; Table 1). CTx-648 is an emerging KAT6i which has shown favorable antitumor activity in vivo in ER+ endocrine-therapy-resistant BCa models [69].

The bromodomain and extraterminal (BET) family of proteins, BRD2, BRD3, BRD4 and the testis-specific BRDT, are readers of acetyl-lysine residues within chromatin and regulate gene expression [70,71]. Numerous studies investigating inhibitors targeting BET proteins have been undertaken in recent years (nicely reviewed in [72]). Preclinical studies found that BET inhibition attenuates AR signaling and is a promising therapeutic approach for CRPC [73].

Birabresib (OTX015) acts as a BRD2, 3 and 4 inhibitor by competitively occupying the acetyl-binding pockets of these proteins [74]. Birabresib has been used in a phase I trial for acute leukemia [74] and for solid tumors in a phase Ib trial (NCT02259114). Within the phase Ib trial, 26 (57%) patients had CRPC and partial response was not observed in any CRPC patients, although stable disease was observed in 2 patients [75]. ZEN003694, a pan-BET inhibitor, is being used in a phase II trial in combination with a PARPi in advanced solid tumors (NCT05327010), as well as in several trials in hormone-dependent cancers (described in Table 2). Recruitment for a phase I trial is ongoing for EP31670 (NEO2734), a dual BET and CBP/p300 inhibitor. This trial includes patients with advanced solid cancer and hematological malignancies including CRPC (NCT05488548, Table 2). NUV-868, another BETi, binds to the BD2 domain of the BRD4 protein, preventing the interaction between BET proteins and acetylated histones [76]. A phase I/II clinical trial of this inhibitor is recruiting to determine its effectiveness as a monotherapy and in combination with olaparib or enzalutamide in OvCa, PCa, BCa and pancreatic cancer (NCT05252390, Table 2). CCS1477, also known as inobrodib, is a selective EP300/CBP bromodomain inhibitor [77]. This inhibitor was initially evaluated in PCa; however, studies are now looking at its benefit in other cancers [78]. Phase I/II clinical trials of this inhibitor are currently recruiting to evaluate it in advanced tumors, including mCRPC and metastatic BCa (NCT03568656, Table 2), and also for hematological malignancies (NCT04068597).

### 2.3. Histone Deacetylase Inhibitors

Histone deacetylase inhibitors (HDACis) block the deacetylase activity of HDAC enzymes, increasing acetylation on various histone and non-histone substrates [79]. HDACs play crucial roles in cancer including the regulation of the cell cycle, DNA damage repair, apoptosis, metastasis and angiogenesis [80,81]. Therefore, a range of HDACis have been developed and tested in different cancer types (Table 1) [81].

Romidepsin (FK228) is an HDACi which targets HDAC class I (HDAC1/2/3/8) and class II (HDAC4/5/6/7/9/10) enzymes and is currently approved by the FDA for the treatment of T-cell lymphoma [82]. The disulfide bonds in the drug are reduced to a thiol group within the cells, which then binds the zinc atom at the active site of the HDACs, inhibiting their activity [83]. The antitumor activity of romidepsin includes the promotion of the p53/p21 signaling pathway, cell cycle arrest, apoptosis, the inhibition of angiogenesis and the modification of the tumor microenvironment [84].

Vorinostat (suberoylanilide hydroxamic acid) targets class I HDAC1/2/3 and HDAC6 from class II, at nanomolar concentrations, and was FDA-approved for the treatment of cutaneous T-cell lymphoma (CTCL) in 2006 [85,86]. The drug binds to the active site of the HDAC enzymes, thus inhibiting their catalytic activity [87]. HDAC inhibition by vorinostat has been shown to suppress the proliferation of cancer cells by inducing cell cycle arrest in vitro and promoting antitumor activity in vivo [85]. Vorinostat inhibition of HDAC activity can also promote acetylation on non-histone proteins, including transcriptional factors (TFs) like AR, p53 and NF-κβ; the mechanism of action is not well understood, but these changes go on to alter the expression of the TF target genes [85,88].

Pracinostat (SB939) is a pan-HDACi, targeting class I, II and IV HDACs. Pracinostat induces the growth arrest of cancer cells at the G0–G1 phase of the cell cycle, along with the activation of TSGs [89,90]. Pracinostat has promising activity in vitro and in vivo, in colorectal, prostate and ovarian cancer models [91,92].

Panobinostat (LBH589) is also a pan-HDACi with broad HDAC- and non-histone deacetylase-inhibitory activity [93]. It showed potent antitumor activity in preclinical studies in PCa models, thus progressing to clinical trials [94]. Panobinostat has also been demonstrated to modulate host innate immune responses, potentially enhancing the effectiveness of the trastuzumab monoclonal antibody in HER2+ BCa [95]. It has also been shown to regulate key oncogenic pathways, like AKT/mTOR signaling and β-catenin, essential for tumor growth and survival [96,97]. Entinostat (SNDX-275 or MS-275) is a benzamide derivative acting as a selective inhibitor of class I HDACs. It can regulate genes associated with angiogenesis in TNBC, upregulating TSGs and anti-angiogenic gene expression and leading to the suppression of metastasis and aggressive tumor characteristics [98]. Entinostat can also work synergistically with CDK4/6 inhibitors to suppress the proliferation of ER+ BCa and TNBC cells [99]. Additionally, entinostat can enhance the presentation of neoantigens, improving the infiltration of CD8+ T lymphocytes in cancer cells [100], which also reprograms tumors to respond better to existing immunotherapies like PD-1 blockers [101].

Tucidinostat (Chidamide) is an oral HDACi, targeting HDAC class I (HDAC1/2/3) as well as class IIb (HDAC10) [102]. It was approved by the China FDA for treatment in relapsed or refractory peripheral T-cell lymphoma in 2014 [103]. The drug works by regulating the BAX/BCL-2 ratio in cells and inducing cell cycle arrest at G0/G1 [104,105]. Quisinostat (JNJ-26481585) is a hydroxamate-derived, second-generation HDACi that targets class I HDACs with high specificity, and shows weak potency for class II HDACs [106]. It induces G0/G1 cell cycle arrest, apoptosis and the upregulation of p21 and E- cadherin as observed in vitro and in vivo [107]. Some antitumor activity, along with better tolerability with an intermittent quisinostat dosage as compared to a continuous schedule, was observed in patients with advanced solid tumors in a phase I study [108]. Belinostat is an antineoplastic HDACi that received FDA approval in 2014 as a monotherapy for relapsed or refractory peripheral T-cell lymphoma after the success of the phase II BELIEF trial [109]. Belinostat also induces cell cycle arrest, apoptosis and p21 upregulation, which was demonstrated in prostate, breast, lung and ovarian cancer cells as well as in vivo studies [110]. Valproic acid is a short-chain fatty acid HDACi that targets HDAC1/2 and is well-tolerated in patients due to its clinical use in treating neurological conditions [111]. Following valproic acid treatment, HDAC2 undergoes proteasome-mediated degradation, inducing apoptosis and G1 cell cycle arrest [112]. Valproic acid treatment in PCa in vivo induces cell cycle arrest and apoptosis and reduces AR expression [113].

### 2.4. PRC2 Inhibitors

EZH2 is the catalytic subunit of PRC2, responsible for the trimethylation of histone 3 lysine 27 (H3K27me3) [114]. Several EZH2 inhibitors (EZH2is) have been developed that block the PRC2-mediated methylation of H3K27 (Table 1). Tazemetostat (EPZ-6438) is a first-in-class, oral EZH2i, FDA-approved for use in adults with relapsed follicular lymphoma and advanced epithelioid sarcoma [115]. Tazemetostat competitively binds the EZH2 SET domain to block its methyltransferase function and prevents the hypertrimethylation of H3K27, reversing the transcriptional silencing of genes by PRC2 [116]. The decreased proliferative effects observed during tazemetostat treatment correlate with a downregulation of MYC and WNT/β-catenin pathways, leading to reduced tumor growth [116,117]. Tazemetostat, which inhibits both Y641N-mutant and wild-type forms of EZH2, has been shown to induce cell cycle arrest and apoptosis in preclinical models of follicular lymphoma [116,117]. Some EZH2 gain-of-function mutations are also inhibited by tazemetostat in follicular lymphomas, including Y646X and A637V [115]. Valemetostat is a dual EZH1/2 inhibitor approved for the treatment of T-cell leukemia/lymphoma and is being investigated for the treatment of tumors characterized by an SW1/SNF mutation [118,119]. The use of dual EZH1/2 inhibitors as opposed to a selective EZH2i, like tazemetostat, was found to have superior effects in decreasing tumor cell proliferation, both in vitro and in vivo [119]. Mevrometostat (PF-06821497) is a highly specific, oral EZH2i with effects against both wild-type and Y641N-mutant EZH2. It is currently being tested for the treatment of PCa in at least three ongoing clinical trials (NCT03460977, NCT06551324 and NCT06551324) [120]. Tulmimetostat (CPI-0209) is a second-generation EZH1/EZH2i which was demonstrated to be up to 50-fold more potent than first-generation EZH2is like tazemetostat, with a lower IC_50_, longer on-target residence time and higher suppression of global H3K27me3 in cancer xenografts [121,122]. Tulmimetostat induced the expression of PRC2 target genes in a dose-dependent manner in patients with advanced solid tumors (NCT04104776, Table 1) [122]. Tulmimetostat also showed preferential EZH2 inhibition in *ARID1A*-mutant chemoresistant tumors in vivo, improving cisplatin response in these tumors [121]. SHR2554 is a small-molecule EZH2i that competitively binds the EZH2 catalytic SET domain. It reduces intracellular H3K27me3 levels and reduces lymphoma cell proliferation [123]. It was shown to have synergistic antitumor effects in combination with other agents like HDACis and anti-PD-L1/TGF-βRII drugs [124,125,126].

Embryonic ectoderm development (EED) inhibitors (EEDi) work by targeting the EED subunit of PRC2 [127]. EED is a scaffolding protein which assembles and stabilizes the PRC2 complex [128]. Therefore, PRC2 activity is disrupted by EED inhibition, subsequently reducing PRC2-mediated H3K27me3, resulting in the reactivation of transcriptionally repressed TSGs [129]. MAK683 is a first-in-class and highly selective allosteric EEDi [130]. MAK683 selectivity was tested against a panel of 23 protein methyltransferases, and the only selectivity observed was for EZH1/2-containing PRC2 [131]. In multiple cancer cell lines including rhabdoid tumor (G401, G402), OvCa (A2780, MCAS) and adenocarcinoma (Hs700T, HeLa), MAK683 treatment resulted in reduced cell proliferation [132]. Furthermore, MAK683 treatment resulted in transcriptional activation of *CDKN2A*/*p16*, SASP factors and extracellular matrix genes, and the induction of a differentiation program, which might be due to PRC2 involvement in embryonic development [132]. ORIC-944 is another selective, allosteric EEDi which binds EED and prevents its interaction with other PRC2 members, inhibiting H3K27me3 activity [133]. ORIC-944 demonstrated tumor growth inhibition in AR-positive, AR-mutant, AR-v7, ADT-responsive and ADT-resistant in vivo PCa models [133].

### 2.5. KDM1A Inhibitors

Lysine demethylase 1A (KDM1A), which has been well studied, has both histone and non-histone targets, which were nicely reviewed by Cai and colleagues, 2024, who also summarized current inhibitors in clinical trials for all cancers [134]. In this review we have focused on the current inhibitors utilized for treating endocrine-related cancers and the emergence of KDM1A dual inhibitors. KDM1A demethylates mono- and dimethyl marks on lysine 4/9 on histone 3 (H3K4me1/2 and H3K9me1/2) and can contribute to transcriptional activation or repression of genes [135]. It is also known to regulate transcription by acting as a coregulator for nuclear receptors including AR and ER [38,136]. Currently, two KDM1A inhibitors (KDM1Ai), pulrodemstat (CC-90011) and seclidemstat (SP-2577), are being tested in endocrine-related cancers. These inhibitors cause a reversible inhibition of KDM1A, which is predicted to produce better clinical outcomes compared to irreversible inhibition (Table 1) [137]. Pulrodemstat is a highly potent, orally active, pyrimidinone-based KDM1Ai which was shown to promote antitumor activity in advanced solid tumors and tumors with neuroendocrine differentiation [137,138]. A phase I trial of pulrodemstat in solid tumors and non-Hodgkin lymphoma showed good tolerability and antitumor activity [139]. Phase II trials are being conducted for pulrodemstat alongside etoposide, cisplatin and nivolumab in advanced cancers (NCT04350463 and NCT03850067). Seclidemstat is a non-competitive KDM1Ai which does not prevent the recruitment and binding of KDM1A to the N-terminal H3, but rather restricts the protein–protein interactions crucial for the epigenetic function of KDM1A by promoting its conformational change [140]. On in vitro testing, endometrial, breast, colorectal and pancreatic cancer cells were sensitive to seclidemstat, and an increase in global H3K9me3 levels was observed after drug treatment in VCaP PCa cells, validating biological activity [140]. There are also several KDM1A dual inhibitors being developed that hold promise for dual-targeting therapies, targeting other epigenetic factors or drivers of cancer, such as EGFR [141,142,143,144].

**Table 2:** Ongoing clinical trials testing epigenetic drugs in endocrine-related cancers.

Clinical Trial ID	Cancer Type	Epigenetic Drug Type	Intervention	Primary Outcome Measure	Status (Patient Enrolment)
**NCT02393794**	Breast cancer	HDACi	Romidepsin and cisplatin combination at different doses, with/without nivolumab	Phase I: Recommended dose of romidepsin with cisplatin for phase II Phase II: Objective response rate according to RECIST v1.1 criteria	Phase I/II Active, not recruiting (51)
**NCT03742245**	Breast cancer	HDACi	Vorinostat and olaparib (PARP inhibitor) combination at different doses	Maximum tolerated dose (MTD) of olaparib and vorinostat combination (16 weeks)	Phase I Recruiting (28)
**NCT00616967**	Breast cancer	HDACi	Carboplatin + paclitaxel with/without vorinostat	Pathological complete response rate	Phase II Active, not recruiting (68)
**NCT01349959**	Breast cancer	HDACi DNMTi	Entinostat + azacitidine	Confirmed response rate (complete or partial) as per RECIST criteria	Phase II Active, not recruiting (58)
**NCT02453620**	Breast cancer and other solid tumors	HDACi	Entinostat + nivolumab + ipilimumab	Safety and tolerability analyzed by AEs, SAEs and laboratory abnormalities	Phase I Active, not recruiting (57)
**NCT02115282**	Breast cancer	HDACi	Exemestane with/without entinostat	Progression-free survival (PFS) and overall survival (OS)	Phase III Active, not recruiting (608)
**NCT06556862**	Breast cancer	HDACi	Dalpiciclib (CDK4/6 inhibitor) + tucidinostat	PFS	Phase II Not yet recruiting (155)
**NCT06547476**	Breast cancer	HDACi	Tucidinostat + Tislelizumab (PD-1 inhibitor)	PFS	Phase II Not yet recruiting (40)
**NCT05633914**	Breast cancer	HDACi	Tucidinostat + nab-paclitaxel	Objective response rate as per RECIST v1.1 criteria	Phase II Recruiting (90)
**NCT05411380**	Breast cancer	HDACi	Tucidinostat + Metronomic Capecitabine + endocrine therapy	Objective response rate as per RECIST v1.1 criteria	Phase II Recruiting (73)
**NCT05335473**	Breast cancer	HDACi	Tucidinostat + Eribulin	DLT and MTD for phase Ib, PFS	Phase I/II Recruiting (87)
**NCT06750848**	Breast cancer	HDACi	Tucidinostat + fulvestrant + angiogenesis inhibitor	Objective response rate as per RECIST v1.1 criteria	Phase II Not yet recruiting (48)
**NCT05983107**	Breast cancer	HDACi	Tucidinostat + endocrine therapy (in PIK3CA-wild-type patients) Everolimus + endocrine therapy (in PIK3CA-mutant patients)	First-stage progression-free survival (PFS1)	Phase II Recruiting (102)
**NCT05890287**	Breast cancer	HDACi	Tucidinostat + exemestane/fulvestrant/letrozole/anastrozole/tamoxifen with/without CDK4/6 inhibitor	PFS	Recruiting (60)
**NCT05808582**	Breast cancer	HDACi	Tucidinostat + fulvestrant	PFS	Phase II Not yet recruiting (60)
**NCT05632848**	Breast cancer	HDACi	Tucidinostat + Zimberelimab (targets PD-1)	Objective response rate as per RECIST v1.1 criteria	Phase II Recruiting (47)
**NCT05586841**	Breast cancer	HDACi	Tucidinostat + Dalpiciclib (after failure of CDK4/6 inhibitor)	MTD of combination treatment	Phase I Not yet recruiting (30)
**NCT05464173**	Breast cancer	HDACi	Tucidinostat + Abemaciclib (CDK4/6 inhibitor) + endocrine therapy	DLT and PFS with combination treatment	Phase I/II Recruiting (44)
**NCT05186545**	Breast cancer	HDACi	Tucidinostat + Surufatinib + fulvestrant	PFS	Phase II Recruiting (63)
**NCT04891068**	Breast cancer	DNMTi	Azacitidine	Change in tumor-infiltrating lymphocyte (TIL) count in primary tumors from patients with high-risk early-stage breast cancer	Phase II Recruiting (40)
**NCT01349959**	Breast cancer	DNMTi HDACi	Azacitidine + entinostat	Confirmed response rate (partial or complete response) as per RECIST criteria	Phase II Completed (58)
**NCT05422794**	Breast cancer	BETi	ZEN003694 + nab-paclitaxel + pembrolizumab (at different doses)	MTD, DLT and recommended phase II dose, incidence of AEs	Phase I Recruiting (57)
**NCT05372640**	Breast cancer	BETi	ZEN003694 + Abemaciclib	MTD and recommended phase II dose, AEs, objective response rate, clinical benefit rate (CBR), duration of response, time to response, OS, PFS	Phase I Recruiting (30)
**NCT05633979**	Breast cancer	EZH2i	Valemetostat with/without trastuzumab deruxtecan	Objective response rate	Phase I Recruiting (37)
**NCT04355858**	Breast cancer	EZH2i	SHR2554 + SHR3680 AR inhibitor (in screened cohort with AR > 10%) SHR2554 + SHR3162 (PARP inhibitor)	Objective response rate as per RECIST v1.1 criteria	Phase II Recruiting (319)
**NCT06145633**	Prostate cancer	HDACi	Vorinostat + 177Lu-prostate-specific membrane antigen [PSMA]-617	Percentage of patients converted from PSMA-low to PSMA-high	Phase II Recruiting (15)
**NCT04703920**	Prostate cancer, breast cancer and ovarian cancer	HDACi	Belinostat + Talazoparib (PARP inhibitor)	DLT within first two cycles	Phase I Completed (26)
**NCT02998567**	Prostate cancer and other solid tumors	DNMTi	Guadecitabine (escalating dose) + pembrolizumab	MTD, AEs according to Common Terminology Criteria for Adverse Events (CTCAE) v4.0	Phase I Recruiting (34)
**NCT05488548**	Prostate cancer and other solid tumors and hematological malignancies	BETi	EP31670 (dose escalation)	MTD, DLT, recommended phase II dose	Phase I Recruiting (75)
**NCT04840589**	Prostate cancer and other solid tumors	BETi	ZEN003694 + nivolumab with/without ipilimumab	MTD, DLT, recommended phase II dose	Phase I Recruiting (66)
**NCT04471974**	Prostate cancer	BETi	ZEN003694 + enzalutamide + pembrolizumab	Complete response rate as per RECIST v1.1	Phase II Recruiting (54)
**NCT04986423**	Prostate cancer	BETi	ZEN003694 + enzalutamide combination versus enzalutamide monotherapy	Radiographic PFS (rPFS) as per RECIST v1.1 criteria	Phase II Recruiting (200)
**NCT03568656**	Prostate cancer and other advanced solid tumors	BETi	CCS1477 monotherapy (dose escalation) CCS1477 + abiraterone acetate (antiandrogen) CCS1477 + enzalutamide (antiandrogen) CCS1477 + Darolutamide (antiandrogen) CCS1477 + olaparib CCS1477 + atezolizumab (PD-L1-targeting drug)	Incidence of AEs and SAEs, laboratory assessments (clinical chemistry and hematology)	Phase I/II Active, not recruiting (350)
**NCT04846478**	Prostate cancer	EZH2i	Tazemetostat + Talazoparib (dose escalation)	Rate of DLT, number of participants with treatment-related AEs as per CTCAE v5.0	Phase I Active, not recruiting (35)
**NCT06632977**	Prostate cancer	EZH2i	Valemetostat (after genetic testing)	Objective response rate as per RECIST v1.1	Phase II Recruiting (474)
**NCT04388852**	Prostate cancer	EZH2i	Valemetostat + ipilimumab	Incidence of AEs, MTD	Phase I Active, not recruiting (65)
**NCT03460977**	Prostate cancer and other solid tumors	EZH2i	Mervometostat (dose escalation) versus mevrometostat + enzalutamide	Percentage patients with DLTs, MDT, AEs, objective response rate as per RECIST v1.1, laboratory abnormalities, changes in vital signs, rPFS	Phase I Recruiting (343)
**NCT06629779**	Prostate cancer	EZH2i	Enzalutamide with/without mevrometostat	rPFS	Phase III Recruiting (900)
**NCT06551324**	Prostate cancer	EZH2i	Mevrometostat + enzalutamide versus docetaxel + enzalutamide	rPFS	Phase III Recruiting (600)
**NCT04104776**	Prostate cancer, ovarian cancer, endometrial cancer and other solid tumors	EZH2i	CPI-0209 monotherapy in cohort of mCRPC patients, cohort of ovarian clear-cell carcinoma patients (with ARID1A mutations), cohort of endometrial carcinoma patients (with ARID1A mutation)	Frequency of DLTs, objective response rate as per RECIST v1.1 criteria	Phase I/II Recruiting (275)
**NCT05413421**	Prostate cancer	EEDi	ORIC-944 monotherapy (dose escalation) ORIC-944 + abiraterone acetate/apalutamide/Darolutamide/enzalutamide	Recommended phase II dose, pharmacokinetic evaluation of single and combination therapy	Phase I Recruiting (250)
**NCT04606446**	Prostate cancer, breast cancer and other advanced solid tumors	KAT6Ai	PF-07248144 monotherapy PF-07248144 + fulvestrant PF-07248144 + letrozole endocrine therapy + Palbociclib (dose escalation and expansion study)	Determine DLTs, AEs, lab abnormalities	Phase I Recruiting (320)
**NCT04315233**	Ovarian cancer and breast cancer	HDACi	Belinostat + Ribociclib (CDK4/6 inhibitor)	Determine MTD and DLT with combination treatment	Phase I Recruiting (34)
**NCT05983276**	Ovarian cancer	DNMTi	Decitabine with carboplatin/paclitaxel/selinexor	Determine safety and AEs of two drugs in combination	Phase II Recruiting (40)
**NCT02650986**	Ovarian cancer and other cancers	DNMTi	Decitabine with/without cyclophosphamide (chemotherapy) and TGFbDNRII-transduced autologous TILs (in tumors expressing cancer–testis antigens 1 -NY-ESO-1)	Number of participants with DLTs and feasibility concerns in manufacturing of NY-ESO-1/dnTGFbetaRII engineered cells	Phase I/II Active, not recruiting (15)
**NCT03206047**	Ovarian cancer	DNMTi	Atezolizumab with/without guadecitabine	Incidence of AEs, PFS	Phase I/II Completed (12)
**NCT03017131**	Ovarian cancer	DNMTi	Decitabine, cyclophosphamide, genetically engineered NY-ESO-1-specific T lymphocytes, Aldesleukin (recombinant interleukin-2)	Incidence of AEs	Phase I Active, not recruiting (9)
**NCT05071937**	Ovarian cancer	BETi	ZEN003694 + Talazoparib	Confirmed response rate as per RECIST v1.1	Phase II Recruiting (33)
**NCT05252390**	Ovarian cancer, prostate cancer, breast cancer and other solid tumors	BETi	NUV-868 monotherapy NUV-868 + olaparib NUV-868 + enzalutamide (prostate cancer)	Incidence of DLTs, pharmacokinetics, objective response rate as per RECIST v1.1, rPFS, PSA50 response rate	Phase I/II Recruiting (82)
**NCT05950464**	Endometrial cancer and ovarian cancer	BETi	ZEN003694 + Tuvusertib (ATR kinase inhibitor)	DLTs, measurement of gammaH2AX, incidence of AEs	Phase I Recruiting (60)
**NCT03348631**	Endometrial cancer and ovarian cancer	EZH2i	Tazemetostat	Objective response rate as per RECIST v1.1	Phase II Active, not recruiting (62)

## 3. Clinical Trials Utilizing Epigenetic Therapeutics in Endocrine-Related Cancers

### 3.1. Breast Cancer

Epigenetic therapies have been widely trialed in breast cancer, especially HDACi. A clinical trial investigating vorinostat with endocrine therapy in stage IV BCa found a 60% clinical response rate (CRR) in 10 patients receiving vorinostat and an anastrozole inhibitor in combination. The duration of response (DOR) was 29.6 weeks in six patients, progression-free survival (PFS) was 2 months in 15 patients, and overall survival (OS) was 19 months. One patient was also alive 55 months after therapy had started (NCT01720602). Vorinostat has also been trialed in stage IV BCa patients receiving aromatase inhibitors, where it was found that the CRR in the eight patients analyzed was 15%. The PFS was 2.8 months and the OS was 28.8 months, indicating some clinical benefit (NCT01153672). A study investigated the use of carboplatin and nab-paclitaxel with vorinostat or a placebo, in newly diagnosed operable BCa. The results showed that 9/31 participants (29%) receiving the placebo and 8/31 participants (25.8%) receiving Vorinstat showed a pathological complete response (pCR), which is defined as no viable invasive cancer in the breast or axilla (NCT00616967). This suggests that the addition of vorinostat did not yield much more benefit. However, cumulative methylation index (CMI) evaluation performed in the study to measure DNA methylation on a panel of 10 genes suggested a potential value of high CMI levels on day 15 of therapy in predicting poor response to treatment [145]. Further investigations looked at the vorinostat-enhanced effectiveness of standard chemotherapy +/− trastuzumab in locally advanced BCa and found that the pCR was the highest in HER2+ patients (54.2%) in the 24 patients analyzed. TNBC patients had a pCR of 26.7% when 15 patients were analyzed, but none of the ER+/HER2- patients demonstrated a pCR (NCT00574587), indicating that the addition of vorinostat to chemotherapy may be beneficial in a subset of BCa patients [146]. A 54-participant study testing a vorinostat, paclitaxel and bevacizumab combination in metastatic BCa found that the overall response rate (ORR) was 49%. The PFS was 11.9 months, and the OS was 29.4 months (NCT00368875). Additionally, increased acetylation on *Hsp90* and *α-tubulin* was observed on vorinostat treatment, consistent with preclinical results, evidencing biological function [147]. Thus, the combination treatment showed decent efficacy. A vorinostat and trastuzumab combination study in patients with metastatic or locally recurrent BCa unfortunately showed no improvement in tumor response, proving the combination therapy to be inefficient in this cohort (NCT00258349) [148]. A study looking at panobinostat and letrozole in metastatic BCa did not find a clinical response in any patients in phase II. The confirmed response rate in phase I was 0/6 with 20 mg panobinostat + 2.5 mg letrozole, compared to 2/6 with 30 mg of panobinostat and 2.5 mg of letrozole (NCT01105312).

A phase II clinical trial investigating azacitidine and entinostat in advanced BCa found the confirmed response rate to be 4/27 in hormone-resistant BCa (HRBC), but no clinical response was observed in TNBC patients. The OS was 6.6 months in 13 TNBC participants and 12.6 months in HRBC, followed up for up to 3 years (NCT01349959). Although slightly more response was observed in HRBC than TNBC, the overall results did not warrant the progression of the trial [149]. A phase II study in advanced TNBC found the ORR to be 12.5% (n = 45) in a group receiving entinostat and atezolizumab, compared to an ORR of 2.4% in a placebo plus atezolizumab group (n = 41). The clinical benefit rate was 15% in the first group compared to 7.3% in the placebo group. The data from this trial suggested that combination therapy was more effective than atezolizumab alone (NCT02708680). A phase III trial investigating exemestane with entinostat (Arm A; n = 180) and without (Arm B; n = 180) in recurrent hormone receptor-positive BCa found the PFS to be similar in both groups (median of 3.3 months vs 3.1 months in Arms A and B, respectively). Both groups also had a similar OS rate (23.4 months in Arm A, n = 305 vs 21.7 months in Arm B, n = 303) and ORR (4.6% in Arm A, n = 305 vs 4.3% in Arm B, n = 303), suggesting that the addition of entinostat to exemestane had a very modest benefit (NCT02115282) [150]. Similarly, the ENCORE301 study, which investigated exemestane with or without entinostat in postmenopausal women with advanced BCa, found the PFS to be 2.27 months in the placebo group (n = 66) and 4.28 months in the combination group (n = 64). The ORR was 4.6% in the control group compared to 4.7% in the treatment group, suggesting no tangible benefit in response to entinostat addition (NCT00676663). Another study investigating the addition of entinostat to aromatase inhibitors in postmenopausal women with ER+ BCa found the clinical benefit rate to be 15.4% in 26 participants. The PFS was 3.9 months and the ORR during the first six cycles of the study’s treatment was 3.9% (NCT00828854).

A phase III study investigated the efficacy of tucidinostat in combination with exemestane, compared to a placebo plus exemestane, in patients with hormone receptor-positive/HER2-negative BCa which had progressed after endocrine therapy (NCT02482753). Better progression-free survival (PFS) was observed with tucidinostat and exemestane (7.4 months) compared to the placebo and exemestane (3.8 months) [151]. This suggested that epigenetic alteration using tucidinostat along with hormone therapy could be effective in patients with recurrent hormone receptor-positive BCa after hormone therapy.

The DNMTi azacitidine was assessed along with abraxane chemotherapy in patients with advanced or metastatic HER2-negative BCa in a phase II trial (NCT00748553). Improvement in the ORR compared to baseline was seen in 53.8% of patients (n = 13). The treatment was well-tolerated in patients, indicating safety and some clinical benefit with azacitidine treatment in this patient group. Neoadjuvant decitabine treatment followed by pembrolizumab immunotherapy was given to HER2-negative BCa patients, which resulted in an absolute increase in tumor-infiltrating lymphocyte (TIL) stromal occupancy by 7.65% in the TNBC cohort and 6% in hormone receptor (HR)-positive tumors (NCT02957968), indicating an enhanced response to immunotherapy after decitabine treatment.

The BETi ZEN003694 sensitized wild-type BRCA1/2 TNBC to a PARPi, and the clinical benefit rate was 35% (18/51) (NCT03901469). The KAT6i PF-07248144 showed antitumor effects, alone and in combination with fulvestrant, in HR^+^/HER2^−^ metastatic BCa patients. However, the objective response rate (ORR) and PFS were better in combination as compared to in the monotherapy group [152]. Ongoing studies are testing the DNMT inhibitor decitabine, BET inhibitor ZEN003694 and EZH2 inhibitors valemetostat and SHR2554 in advanced BCa (summarized in Table 2).

### 3.2. Ovarian Cancer

Current treatment options in OvCa depend on the type and stage of the tumor and any genetic alterations, with a first line of treatment surgery and chemotherapy (platinum-based therapies, such as cisplatin) [153]. In the presence of genetic mutations in genes involved in DNA repair, such as *BRCA1/2* mutations, the use of PARP inhibitors has been introduced, but cases of resistance have arisen [153,154]. Therefore, many of the epigenetic drugs being clinically trialed are being utilized in combination with current therapies for recurring tumors that have acquired resistance (Table 1).

HDACi have been tested as monotherapies and alongside chemotherapies in recurrent platinum-resistant OvCa. A phase II trial investigated vorinostat in 27 patients and identified vorinostat as a monotherapy that showed little efficacy [155]. Reported in 2015, a phase I study combined vorinostat with carboplatin and gemcitabine in recurrent platinum-sensitive OvCa; in the fifteen patients that received the combination therapy, three developed hematological toxicities, and therefore, the trial was terminated [156]. More recently, a phase II trial testing vorinostat in combination with carboplatin and paclitaxel in 55 patients with recurrent platinum-sensitive OvCa showed promise in tolerability and overall survival (median 40.6, 95% CI 25.1–56.1) [157]. A trial combining the HDACi Belinostat and Ribociclib (CDK4/6 inhibitor) is currently underway (NCT04315233).

Hypomethylating agents (HMAs) are currently being trialed in combination therapies to increase the efficacy of the partnered therapy (Table 1). A phase I study by Falchook and colleagues investigated the safety and tolerability of a combination DNMTi and HDACi with carboplatin [158]. The study identified dosage toxicity in 18% of patients (6/32 patients), and the treatment regimen showed little antitumor effect on advanced OvCa. A multi-centered phase II trial examined the use of an HMA, guadecitabine, in combination with carboplatin (NCT01696032). The study found no significant difference between combination therapy compared to carboplatin alone for OFS and median PFS, but a subset of patients showed improvement in 6-month PFS [159]. Other trials underway use BET inhibitors and recently developed EZH2 inhibitors. BET inhibitors are being trialed alongside PARP inhibitors (NCT05071937, NCT05252390), and the EZH2 inhibitor tazemetostat is being trialed as a monotherapy (NCT03348631).

### 3.3. Endometrial Cancer

Since a loss of PR is a common progestin-resistance mechanism in ECa, investigation has been conducted into epigenetic therapies that can reverse the PR downregulation mechanism in ECa [160,161]. The HDACi entinostat when tested in vitro in combination with a progestin, medroxyprogesterone acetate (MPA), showed an increase in the expression of PR protein and its target gene, *PAEP* [161]. In vivo studies with the combination treatment showed a decline in ECa tumor weight and volume compared to the control and MPA alone, but no change in PR expression [161]. Encouraging results from a preclinical study led to the approval of a phase I trial testing entinostat in combination with MPA in patients with endometrioid ECa before hysterectomy (NCT03018249). The primary outcome was the mean PR H-score, which was expected to be higher with combination treatment. Unfortunately, the mean PR H-score was slightly lower in the combination-treatment group (42.7) as compared to the MPA-only group (53.6) [162]. About 70% of patients showed a histological response in the combination group and 72.7% in MPA-only group. However, a higher reduction in Ki-67 expression was observed in the combination group (90%) compared to the MPA-only group (68.2%) [162]. Therefore, limited clinical benefit was observed with the addition of entinostat to MPA treatment in this patient cohort.

Romidepsin, being a more potent HDACi, has shown more promising preclinical results than entinostat. Romidepsin treatment increased the sensitivity of ECa tumors to progestin therapy [161]. Additionally, the reversal of the epigenetic transcriptional repression mechanism of PR was also validated with a romidepsin and MPA combination treatment, leading to the upregulation of PR expression [161]. Thus, romidepsin may represent a better candidate for future trials. A BETi, ZEN003694, and the EZH2 inhibitor, tazemetostat, are also currently in the early phases of clinical trials for the treatment of recurrent ECa (summarized in Table 2).

### 3.4. Prostate Cancer

A study assessing romidepsin treatment in mCRPC patients with no previous exposure to chemotherapy showed minimal clinical improvement, with only 2/35 patients (5.7%) who previously received hormone therapy showing a radiological partial response that lasted over 6 months and a ≥50% decline in PSA, indicating that the treatment may be beneficial in a selected group of CRPC patients (NCT00106418). Additionally, 11 patients (31.4%) showed constitutional or gastrointestinal toxicity to the drug resulting in treatment discontinuation early in the study; thus, the authors suggested altering the dosage for future studies [163]. A phase II study tested pracinostat monotherapy in patients with recurrent CRPC or metastatic PCa (NCT01075308). The treatment was well-tolerated in patients and resulted in a transition from an unfavorable circulating tumor cell (CTC) profile to a favorable one (<5 CTC/7.5 mL blood) in 64% of patients. However, no significant change in the primary outcome, i.e., the PSA response rate, was observed with the treatment, thus preventing the progression of the trial [164]. Vorinostat in combination with ADT was assessed for treating localized PCa before radical prostatectomy (NCT00589472). A pCR at the time of surgery was not observed in any patients; however, a slightly greater number of patients (55.6%) had a localized tumor within the prostate compared to those with extraprostatic extension (44.4%) after the treatment. Another phase II study tested vorinostat monotherapy in patients with metastatic PCa which had progressed after ADT and chemotherapy treatments. The primary outcome measure was PFS at 6 months. However, all 27 patients stopped receiving the therapy before 6 months: 48% due to disease progression, 41% due to toxicity and 11% because of other reasons (NCT00330161). A phase II study explored the safety and efficacy of intravenous (IV) panobinostat in mCRPC patients (NCT00667862). Unfortunately, limited clinical activity was observed, with 11.4% of patients showing PFS but none with a ≥50% decline in PSA at 24 weeks [94]. Another phase II study tested different doses of panobinostat in combination with bicalutamide in patients with recurrent PCa after castration. PFS (6 months) was observed in 42% of patients receiving a 120 mg weekly dose and 19% of patients receiving a 60 mg weekly dose of panobinostat, both with a 50 mg daily dose of bicalutamide. The 9-month patient PFS percentage was 24% with a 120 mg weekly dose and 9% with a 60 mg weekly dose in addition to bicalutamide (NCT00878436), showing some efficacy of the combination therapy in managing recurrent PCa after castration.

Azacitidine was tested with all-trans retinoic acid (ATRA) in a phase II study in patients with recurrent PCa diagnosed based on rising PSA (NCT03572387). No patients showed more than a 30% reduction in PSA from baseline, measured as the primary outcome. However, 33% of patients who received azacitidine + ATRA for 12 weeks and 25% patients who received delayed treatment after a 12-week initial wait exhibited a prolongation of PSA doubling time (PSA-DT) post-treatment. A phase II study investigating azacitidine monotherapy in CRPC patients demonstrated an increase in PSA-DT to ≥3 months in 19/34 (55.8%) patients, with 14 patients showing some level of PSA decline [165]. *LINE-1* methylation in plasma DNA was reduced with azacitidine treatment, which significantly correlated with PSA-DT prolongation [165]. A reduction in *LINE-1* methylation has been observed in preclinical studies with DNMTi treatment [166,167], hinting toward a potential hypomethylation change in tumor-cell DNA. Another phase I/II study tested azacitidine with docetaxel and prednisone in mCRPC patients (NCT00503984). Although no dose-related toxicities were observed in phase I, in phase II, the azacitidine dose was reduced after toxicity-related death in one patient [168]. However, the combination treatment showed a >50% decline in PSA levels from baseline in 10/19 (52.6%) patients. Additionally, out of the 10 patients evaluated, CR was observed in 1 patient, partial response in 2 patients and stable disease in 5 patients [168]. In vitro studies showed that azacitidine treatment in PCa DU145 cells resulted in the demethylation of DNA damage-inducible alpha (*GADD45A*) promoter, increasing GADD45A expression and resensitizing cells to docetaxel treatment [169]. The NCT00503984 trial also showed a significant reduction in methylation levels of *GADD45A* in circulating tumor DNA (ctDNA) from blood plasma in 10 patients, 6 of whom showed a PSA response (60%), whereas no PSA response was observed in the patients with increased *GADD45A* methylation [168], confirming the biological activity of the drug in responsive patients.

The BETi ZEN003694 was tested in combination with enzalutamide in CRPC patients in a phase I/II trial (NCT02711956). Unfortunately, limited clinical activity was observed with a PSA response rate (≥50% decline) observed in 23% of patients from cohort A, receiving 36–96 mg ZEN003694 with 160 mg enzalutamide, and in 4.4% of patients from cohort B, receiving 48–144 mg ZEN003694 with 160 mg enzalutamide. In a phase II trial in metastatic CRPC (mCRPC), the PSA response with ZEN003694 was 27% (NCT04471974).

The EEDi MAK683 underwent a phase I/II trial to evaluate safety and clinical activity in patients with CRPC and ovarian clear-cell carcinoma (NCT02900651) [130]. Unfortunately, no meaningful clinical activity was observed in CRPC or ovarian cancer patients [130]. Enzalutamide-resistant LNCaP PCa cells were more sensitive to the EEDi than parental LNCaP, which was the basis for the recruitment of CRPC patients [130]. However, the limited clinical activity was suggested to be due to the high number of patients receiving prior antineoplastic therapy and subsequent complex resistance mechanisms [130]. Currently underway is a phase1/1b trial evaluating ORIC-944 treatment in metastatic PCa, alone or in combination with AR pathway inhibitors (NCT05413421, Table 2).

The KDM1Ai pulrodemstat and seclidemstat have also been tested in clinical trials for PCa treatment; however, the results are not yet available (NCT04628988, NCT03895684). Ongoing PCa studies with the above-discussed drugs, alone or in combination with other treatments, are discussed in Table 1. Other ongoing studies in PCa are testing the EZH2is tazemetostat, valemetostat, tulmimetostat and mevrometostat, and the KAT6i PF-07248144 (also summarized in Table 2).

### 3.5. Thyroid Cancer

In vitro, romidepsin has been shown to promote the re-expression of NIS, Tg and TPO in TCa in poorly differentiated papillary and anaplastic TCa cells [170,171]. Vorinostat, panobinostat and valproic acid (VPA) treatment reduced papillary and follicular TCa cell viability and increased radioiodine uptake by upregulating NIS expression [172,173,174]. Despite good preclinical results, unfortunately, HDACi monotherapy has shown limited effectiveness in clinical trials. Initial results from a phase II study testing romidepsin in patients with recurrent or metastatic TCa resistant to RAI showed stable disease up to 8 weeks from the start of treatment in 10 of the 11 patients analyzed (NCT00098813). However, mortality due to grade 4–5 serious adverse events (SAEs) was observed in 2 patients and 12/20 patients suffered SAEs of other grades. Similarly, no objective response was observed with panobinostat monotherapy in metastatic TCa patients; however, 6 of the total 13 patients suffered an SAE (NCT01013597). Thus, some safety concerns were associated with romidepsin and panobinostat treatment in TCa. A phase II study assessing vorinostat in patients with locally advanced or metastatic TCa (NCT00134043) showed a better safety profile in TCa patients compared to romidepsin and panobinostat, but unfortunately showed no tumor response [175]. Similarly, VPA monotherapy failed to demonstrate an objective response or improvement in RAI uptake by tumor cells in metastatic TCa patients [176].

Interestingly, romidepsin treatment in p53-null anaplastic TCa cells induced functional p53 expression, resensitizing these cells to doxorubicin chemotherapy [177]. Thus, romidepsin and doxorubicin combination treatment in TCa patients with p53-null tumors could be a potential strategy for future trials, although alterations in the romidepsin dosage and schedule may be required to limit SAEs.

Decitabine was tested in RAI-resistant TCa in a phase II trial to restore RAI uptake (NCT00085293). However, no improvement in the uptake was observed with treatment, abrogating the progression of the study. Overall, monotherapy with epigenetic drugs in TCa has been unsuccessful at eliciting a therapeutic response. At the time of writing, we could not find any ongoing trials with epigenetic drugs in TCa. However, alternative therapeutic strategies comprising combinations of epigenetic drugs with other treatments like immunotherapy, chemotherapy or protein kinase inhibitors, in targeted TCa patient cohorts, could be tested in future trials, as they have shown promise in preclinical studies [177,178,179,180].

## 4. Conclusions

In the past few decades, numerous epigenetic drugs have been developed and have shown promising antitumor effects and the reversal of treatment resistance in endocrine-related cancers during preclinical studies. Therefore, many of these have quickly progressed to clinical testing in patients.

Unfortunately, non-specificity is an issue with epigenetic drugs due to the complex nature of epigenetic regulation. Many epigenetic regulators and associated modifications work synergistically or antagonistically to regulate gene expression. Thus, targeting one regulator can lead to global changes in the epigenetic landscape within cells, leading to undesired consequences [181]. Therefore, a deeper understanding of the histone code and protein complexes involved in epigenetic regulation is essential to develop more specific therapies. Recent advances in precision medicine have resulted in the emergence of more-targeted therapeutic approaches tailored to patients’ genetic and epigenetic profiles. Such approaches use advanced technologies like next-generation sequencing and whole-genome bisulfite sequencing to analyze epigenetic patterns specific to cancer cells that can aid the identification of more-specific therapeutic targets that maximize therapeutic efficacy and reduce toxicity [182,183].

Ongoing clinical studies are also focusing on the use of epigenetic therapies in combination with conventional cancer therapies, as summarized in Table 2. This approach has shown promise as epigenetic drugs can modulate metabolic characteristics and the cell cycle, improve antigen presentation and alter the TME in cancer cells, increasing the efficacy of chemotherapies and immunotherapies [184]. Additionally, in hormone-dependent cancers, epigenetic drugs are being investigated with existing endocrine therapies, as preclinical results have strongly suggested that epigenetic drugs can resensitize resistant tumors to treatments like selective ER modulators, ADT, RAI and chemotherapy [185,186].

This review has presented the integration of epigenetic therapies with conventional cancer treatments. Targeted precision medicine approaches present promising opportunities to enhance the therapeutic efficacy of epigenetic drugs, overcome treatment resistance and improve patient outcomes for endocrine-related cancers.

## Figures and Tables

**Table 1:** Summary of epigenetic drugs and their mechanism of action.

Epigenetic Drug Type	Inhibitor	Mechanism of Action
DNMTi	Azacitidine	Forms covalent adducts with DNMTs, trapping them and preventing DNA methylation.
	Decitabine	Covalently binds DNMTs, depleting their activity.
	Guadecitabine	Cleavage of phosphodiester bond, leading to gradual decitabine release. Resistant to degradation by cytidine deaminase unlike decitabine.
	Hydralazine	Binds the active site of DNMT1 and DNMT3A, inactivating their methyltransferase activity.
	GSK3685032	Reversible inhibition of DNMT1 activity.
	GSK3484862	Leads to DNMT1 degradation through a proteasome-dependent mechanism.
KAT6i	PF-07248144	Inhibits the catalytic activity of KAT6A and KAT6B, modulating global H3K23ac levels. Exact mechanism of action unknown.
	CTx-648	
BETi	Birabresib	Binds to the acetyl-binding pockets of BRD2, BRD3 and BRD4, inhibiting their activity.
	ZEN003694	Pan-BET inhibitor.
	EP31670	Binds to BET proteins (BRD2, BRD3, BRD4 and BRDT) and the bromodomains of CBP/p300, inhibiting their activity.
	NUV-868	Binds to the BD2 domain of BRD4, preventing interaction between BET proteins and acetylated histones.
	CCS1477	Selectively inhibits EP300/CBP bromodomain.
HDACi	Romidepsin	Inhibits HDAC class I and class II enzymes by binding to the zinc atom on the active site of the enzymes.
	Vorinostat	Inhibits HDAC1/2/3 from class I and HDAC6 from class II by binding to the active site of the enzymes.
	Pracinostat	Pan-HDAC inhibitor.
	Panobinostat	Pan-HDAC inhibitor. Inhibits deacetylation on histone and non-histone targets.
	Entinostat	Selective HDAC class I inhibitor.
	Tucidinostat	Targets HDAC1/2/3 from class I and HDAC10 from class IIb.
	Quisinostat	Targets class I HDACs and exhibits weak potency for class II HDACs.
	Belinostat	Pan-HDAC inhibitor.
	Valproic acid	Proteasome-mediated degradation of HDAC2.
EZH2i	Tazemetostat	Competitively binds to the EZH2 SET domain and inhibits EZH2 methyltransferase activity. Inhibits Y641N-mutant and wild-type forms of EZH2.
	Valemetostat	Dual EZH1/2 inhibitor. Exact mechanism of action unknown.
	Mevrometostat	Inhibits Y641N-mutant and wild-type EZH2, reducing H3K27me3 levels, but exact mechanism of action unknown.
	Tulmimetostat	Second-generation EZH1/2 inhibitor, reduces global H3K27me3 levels.
	SHR2554	Competitively binds to the EZH2 SET domain and inhibits EZH2 methyltransferase activity.
EEDi	MAK683	Binds to EED and disrupts EED-EZH1/2 interaction, allosterically inhibiting PRC2 activity.
	ORIC-944	Allosteric inhibition of PRC2 activity by binding to EED.
KDM1Ai	Pulrodemstat	Binds to the AO pocket of KDM1A, resulting in reversible inhibition of KDM1A activity.
	Seclidemstat	Non-competitive KDM1A inhibition by blocking the enzymatic and scaffolding activity of KDM1A.

## Abbreviations

The following abbreviations are used in this manuscript:

ADT	Androgen deprivation therapy
AE	Adverse event
AKT	Ak strain transforming
AR	Androgen receptor
ARID1A	AT-rich interaction domain 1A
ASH2L	ASH2 like, histone lysine methyltransferase complex subunit
ATRA	All-trans retinoic acid
BAX	Bcl2-associated X
BCa	Breast cancer
BCL2	B-cell lymphoma 2
BET	Bromodomain and extraterminal
BETi	Bromodomain and extraterminal inhibitor
BMP7	Bone Morphogenetic Protein 7
BRCA1/2	Breast cancer gene 1 or 2
BRD	Bromodomain-containing
BRDT	Bromodomain testis-specific protein
CBP	CREB-binding protein
CBR	Clinical benefit rate
CDH1	Cadherin 1
CDK4/6	Cyclin-dependent kinase 4/6
CDKN2A	Cyclin-dependent kinase inhibitor 2A
CR	Complete response
CRPC	Castration-resistant prostate cancer
CRR	Clinical response rate
CSC	Cancer stem cell
CTC	Circulating tumor cell
CTCAE	Common Terminology Criteria for Adverse Events
CTCL	Cutaneous T-cell lymphoma
CtDNA	Circulating tumor DNA
DLT	Dose-limiting toxicity
DNA	Deoxyribonucleic acid
DNMT	DNA methyltransferase
DNMTi	DNA methyltransferase inhibitor
DOR	Duration of response
ECa	Endometrial cancer
EED	Embryonic ectoderm development
EEDi	Embryonic ectoderm development inhibitor
EGFR	Epidermal growth factor receptor
EHMT2	Euchromatic histone lysine N-methyltransferase 2
ER	Estrogen receptor (protein)
ESR1	Estrogen receptor 1 (gene)
EZH2	Enhancer of zeste homolog 2
EZH2i	Enhancer of zeste homolog 2 inhibitor
FDA	Food and Drug Administration
GADD45A	DNA damage-inducible alpha
GSTP1	Glutathione S-transferase Pi 1
H3K23ac	Histone 3 lysine 23 acetylation
H3K27me3	Histone 3 lysine 27 trimethylation
H3K4me2/3	Histone 3 lysine 4 di-/trimethylation
H3K9me	Histone 3 lysine 9 methylation
HAT	Histone acetyltransferase
HDAC	Histone deacetylase
HDACi	Histone deacetylase inhibitor
HER2	Human epidermal growth factor receptor 2
HMA	Hypomethylating agent
HOXD3	Homeobox D3
HR	Hormone receptor
HRBC	Hormone-resistant breast cancer
Hsp90	Heat shock protein 90 (gene)
KAT6A/B	Lysine acetyltransferase 6 A/B
KAT6i	Lysine acetyltransferase 6 inhibitor
KDM	Lysine demethylase
KDM1A	Lysine demethylase 1A
KDM1Ai	Lysine demethylase 1A inhibitor
KLK3	Kallikrein-related peptidase 3
KMT	Lysine methyltransferase
LINE-1	Long Interspersed Nuclear Element-1
LSD1	Lysine-specific demethylase 1
mCRPC	Metastatic castration-resistant prostate cancer
MGMT	O6-methylguanine-DNA methyltransferase
MLH1	MutL homolog 1
MPA	Medroxyprogesterone acetate
MTD	Maximum tolerated dose
mTOR	Mechanistic target of rapamycin kinase
NF-κβ	Nuclear factor kappa-light-chain-enhancer of activated B cells
NIS	Sodium iodide symporter
NSD2	Nuclear Receptor Binding SET Domain Protein 2
ORR	Overall response rate
OS	Overall survival
OvCa	Ovarian cancer
P53	Tumor protein 53
PAEP	Progestagen-Associated Endometrial Protein
PARP	Poly (ADP-ribose) polymerase
PCa	Prostate cancer
pCR	Pathological complete response
PD-1	Programmed cell death 1
PD-L1	Programmed Death-Ligand 1
PFS	Progression-free survival
PFS1	First-stage progression-free survival
PGR	Progesterone receptor (gene)
PIK3CA	Phosphatidylinositol-4,5-bisphosphate 3-kinase catalytic subunit alpha
PR	Progesterone receptor (protein)
PRC2	Polycomb repressive complex 2
PSA	Prostate-specific antigen
PSA-DT	PSA doubling time
PSMA	Prostate-specific membrane antigen
PTEN	Phosphatase and TENsin homolog deleted on chromosome 10
RAI	Radioactive-iodine therapy
RARβ	Retinoic acid receptor β
RASSF1A	Ras association domain family protein 1 isoform A
RECIST	Response Evaluation Criteria in Solid Tumors
RNA	Ribonucleic acid
rPFS	Radiographic PFS
RUNX3	Runt-related transcription factor 3
SAE	Serious adverse event
SASP	Senescence-Associated Secretory Phenotype
SERM	Selective Estrogen Receptor Modulator
SET	Su(var)3–9, Enhancer of zeste, and Trithorax
SLC5A8	Solute carrier family 5 member 8
SWF/SNF	Switch/Sucrose Non-Fermentable
TCa	Thyroid cancer
Tg	Thyroglobulin
TGF-βRII	Transforming growth factor beta receptor type II
TH	Thyroid hormone
TIL	Tumor-infiltrating lymphocyte
TME	Tumor microenvironment
TNBC	Triple-negative breast cancer
TPO	Thyroperoxidase
TSG	Tumor suppressor gene
TSH	Thyroid stimulating hormone
TSHR	TSH receptor
TTF-1	Thyroid transcription factor-1
VPA	Valproic acid
ZEB1	Zinc Finger E-box-Binding Homeobox 1
